# Breast cancer brain metastases genomic profiling identifies alterations targetable by immune-checkpoint and PARP inhibitors

**DOI:** 10.1038/s41698-024-00761-0

**Published:** 2024-12-20

**Authors:** A. Giannoudis, E. S. Sokol, T. Bhogal, S. H. Ramkissoon, E. D. Razis, R. Bartsch, J. A. Shaw, K. McGregor, Alison Clark, R.S.P. Huang, C. Palmieri

**Affiliations:** 1 Institute of Systems, Molecular and Integrative Biology, Molecular and Clinical Cancer Medicine, University of Liverpool, Liverpool, UK; 2 Foundation Medicine, Inc., Boston, MA USA; 3 The Clatterbridge Cancer Centre NHS Foundation Trust, Liverpool, UK; 4 Hygeia Hospital, 3rd Oncology Department, Marousi, Athens Greece; 5 Medical University of Vienna, Department of Medicine I, Division of Oncology, Vienna, Austria; 6 Leicester Cancer Research Centre, Department of Genetics and Genome Biology, University of Leicester, Leicester, UK

**Keywords:** Breast cancer, Cancer genomics

## Abstract

Understanding the genomic landscape of breast cancer brain metastases (BCBMs) is key to developing targeted treatments. In this study, targetable genomic profiling was performed on 822 BCBMs, 11,988 local breast cancer (BC) biopsies and 15,516 non-central nervous system (N-CNS) metastases (all unpaired samples) collected during the course of routine clinical care by Foundation Medicine Inc (Boston, MA). Clinically relevant genomic alterations were significantly enriched in BCBMs compared to local BCs and N-CNS metastases. Homologous recombination deficiency as measured by *BRCA1/2* alteration prevalence and loss-of-heterozygosity and immune checkpoint inhibitor (ICI) biomarkers [Tumor mutation burden (TMB)-High, Microsatellite instability (MSI)-High, *PD-L1/L2*)] were significantly more prevalent in BCBM than local BC and N-CNS. High PD-L1 protein expression was observed in ER-negative/HER2-negative BCBMs (48.3% vs 50.0% in local BCs, 21.4% in N-CNS). Our data highlights that a high proportion of BCBMs are potentially amenable to treatment with targeted therapeutic agents including PARP inhibitors and ICIs.

## Introduction

Breast cancer (BC) is the second most common solid malignancy to involve the central nervous system (CNS)^1^. BC is a heterogeneous disease classified into clinically-relevant subtypes by immunohistochemistry (IHC) and fluorescent in situ hybridization (FISH) detection of estrogen (ER) and progesterone (PR), configuring the hormone receptor (HR)-positive breast cancers, and human epidermal growth factor receptor 2 (HER2) receptors or ERBB2 gene amplification as HER2-positive breast cancers, or lastly the lack of these receptors’ expression, categorizing the triple-negative breast cancers (TNBC)^2–4^. In the metastatic setting, the predilection for distant organs’ involvement including the brain varies by subtype^5^. Breast cancer brain metastases (BCBM) occur more frequently in patients with HER2-positive breast cancer and TNBC; these two sub-types exhibit a 2 to 5-fold increased risk of developing BCBM when compared to HR-positive cancers^5–7^.

BCBMs are an increasingly common clinical scenario in patients living with metastatic breast cancer (MBC), and the development of new onset or progressive brain metastases in the setting of extracranial disease that is adequately controlled is a particular challenge^8^. Active systemic therapeutic options following failure of local CNS therapies are relatively limited, particularly in patients with HER2-negative tumors^5–8^. Given this and the associated morbidity and mortality associated with BCBM, they remain an area of unmet clinical need^6,7^. Patients with active BCBMs are often explicitly excluded from clinical trials, not only disadvantaging them in terms of accessing novel agents but also limiting the development and so availability of novel therapies for these patients^9^. The application of next generation sequencing technologies has enabled the characterization of BCs and has also demonstrated the complex and diverse molecular landscapes of metastatic BCs^7,10–13^. BCBM sequencing studies have identified differences in their genomic landscape as compared to primary tumors^14–17^. Utilizing data from 13 studies, which had sequenced BCBMs, we have previously reported the commonly mutated genes identified in BCBM and how they differed as compared to data from two large studies of extra cranial disease, which lacked any BCBMs^17^. That work emphasized the importance of considering BCBMs as a distinct entity at the genomic level and that data from extracranial disease cannot be reliably utilized. The study highlighted further the importance of profiling the brain metastases to identify appropriate therapeutic options^17^.

Given the lack of representation of BCBM in published datasets of metastatic breast cancer and the overall paucity of available genomic BCBM data^17^, the present study examined the genomic landscape of one of the largest cohorts of BCBMs available. We use it to compare BCBMs to data from a cohort of unpaired local BCs and non-CNS metastases (N-CNS) to determine what potentially actionable targets may be present in BCBMs with the overarching aim being to define what therapies should be rationally considered for clinical development for BCBMs.

## Results

### Genomic profile of breast cancer brain metastases

**Fig. 1:**
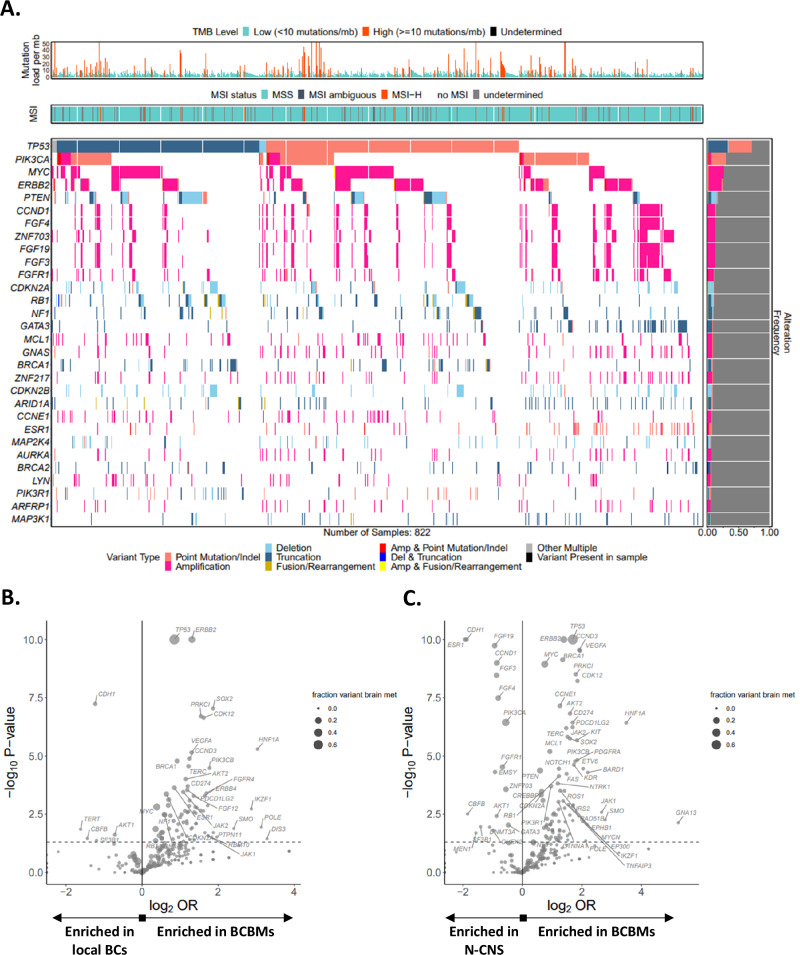
Genomic profile of breast cancer brain metastases. **A** The 30 most frequently altered genes identified in 822 breast cancer brain metastases (BCBM) are included in the tile-plot. Volcano plots comparing the frequency of gene alterations in BCBMs with (**B**) local breast cancers (BCs) and (**C**) non-central nervous system (N-CNS) metastases. *P*-values and odds ratios (OR) were calculated using the Fisher’s exact test.

**Fig. 2:**
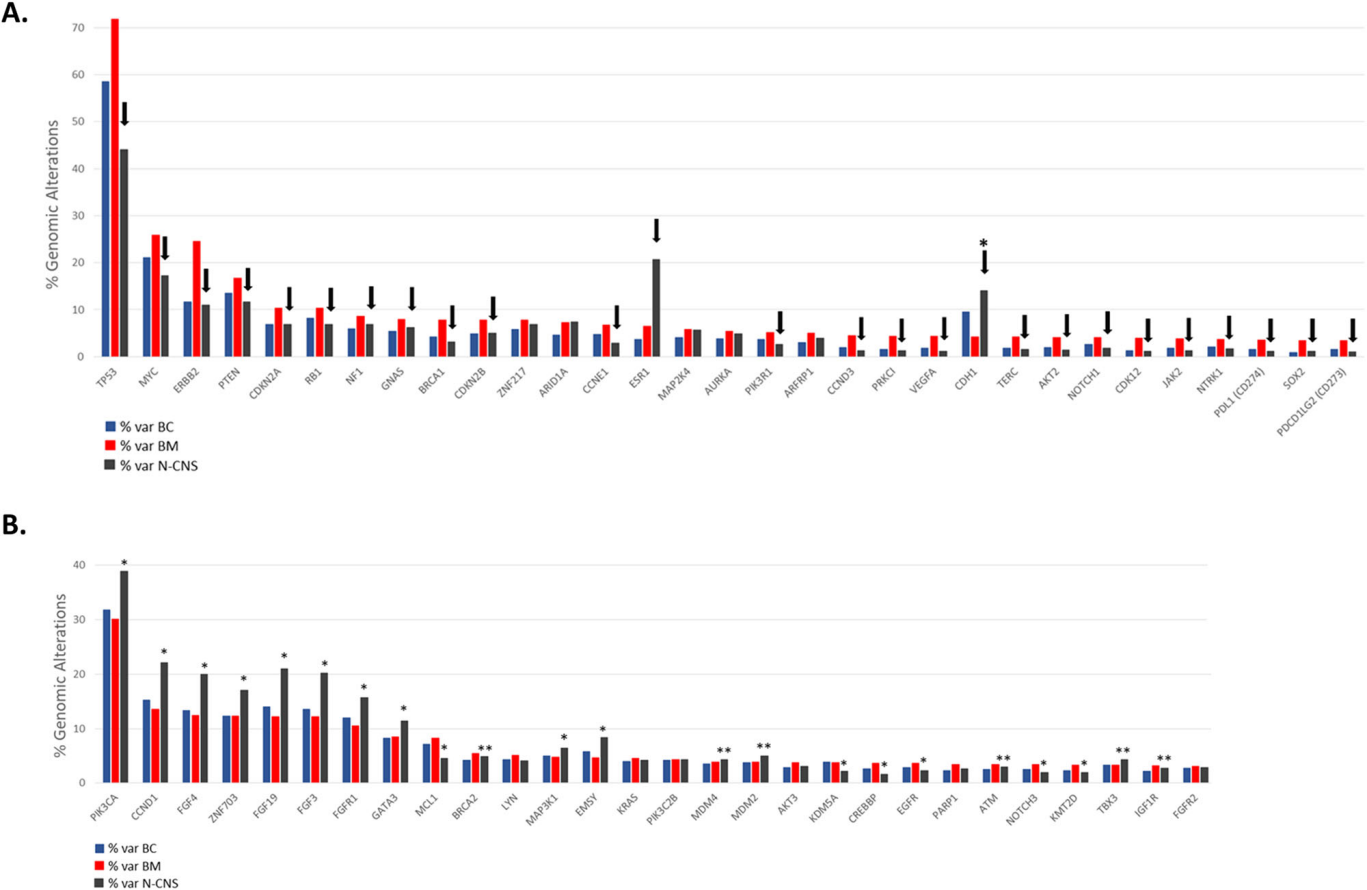
Bar-charts of the genes with significantly different alteration prevalence between BCBM, local breast cancer (BC) and non-central nervous system (N-CNS) metastases. **A** Compared to local BCs, 31 genes were significantly enriched for genomic alterations in BCBMs with prevalence >3% and FDR < 0.1, 26 of them (indicated by the arrows), were also enriched between BCBM vs N-CNS. CDH1 (*) was the only gene with significantly reduced prevalence in BMs as compared to BCs and N-CNS. **B** 28 genes were altered in BCBM with prevalence >3% but not significantly different to local BCs (FDR > 0.1). However, 16 of these genes (indicated by *) were significantly altered in N-CNS as compared to both BCs and BCBMs and 6 genes were significantly altered in N-CNS (indicated by **) as compared to BCs only.

The landscape of genomic alterations in BCBM (*n* = 822) as compared to unpaired local BCs (*n* = 11,988) and N-CNS (*n* = 15,516) metastases is presented in Supplementary Data 1 (Prevalence all). The 30 most frequently altered genes in BCBM including the types of alterations are presented in Fig. 1A whereas the frequent genomic alterations in BCBMs compared to local BCs (Fig. 1B) and N-CNS metastases (Fig. 1C) are highlighted in the volcano plots using the log2 odds ratio (OR) and the adjusted *p*-values. As compared to local BCs, 31 genes with prevalence >3% were significantly different in their alteration prevalence in BCBMs with a false-discovery rate (FDR) < 0.1 and 26 of them (Fig. 2A) were also enriched between BCBM and N-CNS. The most significantly enriched genes in BCBM were: *TP53* (71.8%), *MYC* (25.9%), *ERBB2* (24.6%), *PTEN* (16.7%), *CDKN2A* (10.3%), *BRCA1* and *CDKN2B* (7.8% each), [Supplementary Data 1 (FDR prev all), Fig. 2A]. *ESR1* alterations were more prevalent in BCBM compared to local BCs but both were significantly less than in N-CNS metastases (6.4% vs 3.7%, FDR 0.003 and 20.7%, FDR 6.7 ×10^−27^ respectively). There was no significant difference in *PIK3CA* prevalence between BCBMs (30.2%) and BCs (31.8%) (Fig. 2B). However, *PIK3CA* alterations were more prevalent in N-CNS metastases (38.9%) as compared to both BCs (FDR 5.8 ×10^−33^) and BCBMs (FDR 3.8 ×10^−6^). Overall, 28 genes were altered in BCBM with prevalence >3% but not significantly different to local BCs (FDR > 0.1, Fig. 2B). Several rarer, but potentially actionable alterations were also enriched in the BCBM cohort including alterations in *ROS1*, *KIT*, *NTRK1*, *RICTOR1*, and *JAK2*, each representing ~2-3% of BCBM [Fig. 1B, Supplementary Data 1 (FDR prev all)].

When examining pathogenic short variants (SVs), the most significantly enriched genes in BCBMs relative to local BCs with an FDR < 0.1 were *TP53* (70.7%), *PIK3CA* (26.5%), *BRCA1* (6.7%), *ARID1A* (6.4%), *NF1* (6.1%), *BRCA2* (5.1%) (Table 1), whereas *CDH1* (3.8%) and *AKT1* (1.8%) had significantly reduced prevalence in BCBM in relation to local BCs (Table 1). When examining copy number (CN) alterations, 19 genes had a CN alteration prevalence of at least 3% in BCBM and an FDR < 0.1 compared to local BCs (Table 1). The reported CN alterations represent amplifications (CN > 6) for oncogenes and deep deletions (CN = 0) for tumor suppressors. The top significantly amplified and deleted genes were *MYC* (25.9%), *ERBB2* (22.5%), *PIK3CA* (5.23%) and *PTEN* (9.4%), *CDKN2A* (8.4%), *CDKN2B* (7.4%) respectively. In addition, *PDL1*, *PDCD1LG2* (*PDL2*) and the stemness marker *SOX2* showed a higher amplification prevalence in BCBM at 3.4% compared to 1.6%-0.96% in BC and 1.1%-1.15% in N-CNS for the 3 genes (Table 1). Finally, REs (rearrangements, large structural aberrations) were uncommon genomic alterations in BCBMs. The only gene with an RE prevalence >3% in BCBMs and FDR < 0.1 was *CDK12* (3.5%) (Table 1). All the data of genes showing SVs, CNs and REs in the 3 cohorts is presented in Supplementary Data 2 (Detailed prevalence).

**Table 1:** Detailed prevalence analysis

Gene	% variation in local BC	% variation in BCBM	% variation in N-CNS	FDR BCBM vs BC	FDR BCBM vs N-CNS	FDR N-CNS vs BC
**Short variations**						
TP53_SV	57.7%	70.7%	42.9%	**1.04E−11**	**2.02E−53**	**5.23E−130**
PIK3CA_SV	30.6%	26.5%	38.1%	**6.41E−02**	**2.53E−10**	**2.01E−36**
BRCA1_SV	3.4%	6.7%	2.5%	**1.35E−04**	**1.58E−08**	**3.73E−04**
ARID1A_SV	4.3%	6.4%	6.9%	**4.55E−02**	8.01E−01	**1.39E−19**
NF1_SV	3.9%	6.1%	4.9%	**2.87E−02**	2.64E−01	**1.39E−04**
BRCA2_SV	3.6%	5.1%	4.2%	**1.02E−01**	3.53E−01	**2.71E−02**
ESR1_SV	2.2%	4.7%	19.0%	**7.97E−04**	**2.47E−30**	0.00E+00
CDH1_SV^a^	9.1%	3.8%	13.5%	**6.54E−07**	**1.16E−18**	**2.10E−29**
AKT1_SV^a^	3.7%	1.8%	4.4%	**2.93E−02**	**2.20E−03**	**2.72E−02**
**Copy number alterations**						
MYC_CN	20.98%	25.91%	17.09%	**5.82E−03**	**1.32E−08**	**3.12E−15**
ERBB2_CN	9.18%	22.51%	7.70%	**1.20E−25**	**3.75E−35**	**4.91E−05**
PTEN_CN	5.33%	9.37%	4.73%	**1.24E−04**	**6.27E−07**	**4.45E−02**
CDKN2A_CN	5.34%	8.39%	5.37%	**3.00E−03**	**1.62E−03**	9.44E−01
CDKN2B_CN	4.81%	7.42%	4.86%	**7.50E−03**	**5.05E−03**	8.89E−01
PIK3CA_CN	2.16%	5.23%	2.20%	**2.71E−05**	**7.70E−06**	8.68E−01
ARFRP1_CN	3.02%	4.99%	4.01%	**1.34E−02**	2.36E−01	**4.91E−05**
RB1_CN	2.72%	4.62%	2.04%	**1.22E−02**	**5.17E−05**	**8.48E−04**
CCND3_CN	1.94%	4.50%	1.19%	**1.74E−04**	**5.18E−09**	**3.48E−06**
PRKCI_CN	1.51%	4.38%	1.29%	**4.33E−06**	**3.69E−08**	1.91E−01
VEGFA_CN	1.83%	4.38%	1.17%	**1.24E−04**	**6.05E−09**	**3.46E−05**
TERC_CN	1.87%	4.26%	1.58%	**3.63E−04**	**4.49E−06**	1.08E−01
MAP2K4_CN	1.95%	4.01%	2.82%	**2.82E−03**	**9.52E−02**	**2.00E−05**
AKT2_CN	1.89%	3.89%	1.30%	**2.95E−03**	**2.07E−06**	**4.61E−04**
JAK2_CN	1.73%	3.77%	1.21%	**1.56E−03**	**1.58E−06**	**1.31E−03**
NTRK1_CN	1.89%	3.53%	1.45%	**1.11E−02**	**1.46E−04**	**1.08E−02**
SOX2_CN	0.96%	3.41%	1.15%	**4.33E−06**	**1.04E−05**	1.84E−01
PDCD1LG2_CN	1.53%	3.41%	1.07%	**2.75E−03**	**3.53E−06**	**2.56E−03**
CD274_CN	1.57%	3.41%	1.11%	**2.91E−03**	**6.42E−06**	**3.46E−03**
**Large structural rearrangements**						
CDK12_RE	0.86%	3.53%	0.68%	**7.09E−08**	**3.69E−10**	1.53E−01

The most prevalent genes showing short variations (SV), copy number (CN) alterations (CNAs) and large structural rearrangements (RE) in BCBMs vs local BCs and N-CNS metastases with the corrected *p*-value (FDR) significance between the three cohorts in bold.

^a^Reduced prevalence in BCBM.

### Genomic profile of breast cancer brain metastases according to receptor status

**Fig. 3:**
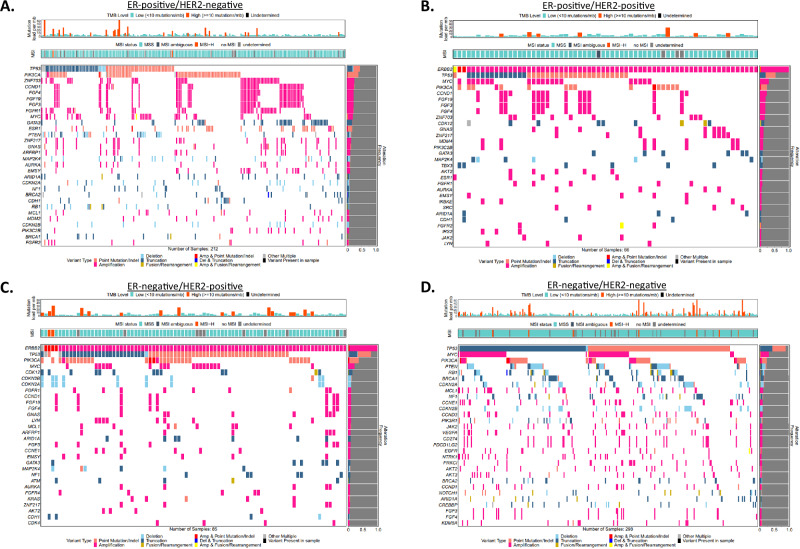
Genomic profile of breast cancer brain metastases according to receptor status. Tile-plots of genomic alterations in BCBMs classified as **A**. ER + /HER2-, **B**. ER + /HER2 + , **C**. ER-/HER2+ and **D**. ER-/HER2-. The different types of genomic variation are indicated by different colors. MSI and TMB for each subtype are also included in the plots.

Genomic profile of BCBMs according to receptor status and comparison of alteration frequency to local BCs and N-CNS metastases was assessed after stratifying all the samples by receptor status or lack of it. *ESR1* and *ERBB2* alterations were as expected, more prevalent within ER-positive and HER2-positive tumors respectively, with *ESR1* alterations (point mutations and amplifications) significantly enriched in ER-positive /HER2-negative BCBMs (FDR < 0.001). The most frequently altered genes in BCBMs such as *TP53*, *PIK3CA*, *MYC*, were present within all the BCBM subtypes. The FGF/FGFR (FGF3/4/19 and FGFR1/2) pathway was frequently altered in ER-positive/HER2-negative (21.7–22.2%), ER-positive/HER2-positive (7.6–19.7%) and ER-negative/HER2-positive (8.2–10.6%) but not in ER-negative/HER2-negative BCBMs. Frequently altered genes by BCBM subtype in ER-positive /HER2-negative cases were *CDH1* (8%) and *BRCA2* (7%); in ER-positive/HER2-positive cases, *PIK3C2B* (11%), *MDM4* (11%), *TBX3* (9%) and *AKT2* (8%); in ER-negative/HER2-positive cases, *LYN* (9%). CDK12 was enriched in HER2-positive BCBMs (15%) compared to local BCs (8.6%) and N-CNS (8.5%) but without reaching significance (*p* < 0.01, FDR > 0.1). Significantly enriched genes FDR < 0.1 by BCBM subtype in ER-negative/HER2-negative were *BRCA1* (14%), *CCND3* (9%), *JAK2* (8%) and the immune checkpoint inhibition (ICI) marker *CD274* (*PDL1*) (7%). The 30 most frequently altered genes according to receptor status are visualized in Fig. 3 and Supplementary Fig. 1 and all the data is available in Supplementary Data 1.

### Genomic signatures and PD-L1 expression of breast cancer brain metastases

We identified significantly higher homologous recombination deficiency as detected by genome-wide loss of heterozygosity (HRD-gLOH; cutoff 16%)^18^, in BCBMs compared to local BCs and N-CNS (52.0% vs 33.7% vs 31.5% respectively) with *p* = 0.0001 for both comparisons (BCBM vs BC and BCBM vs N-CNS) (Table 2). HRD-gLOH was particularly high in the ER-positive/HER2-negative (43.2%, *p* = 0.0682 vs BC and *p* = 0.0306 vs N-CNS) and ER-negative/HER2-negative (70.5%, *p* = 0.0269 vs BC and *p* = 0.0039 vs N-CNS) BCBMs compared to both BCs and N-CNS (Table 2). Consistent with this, alterations in *BRCA1, BRCA2* and *PALB2* were more prevalent in BCBM (7.8%, 5.5% and 2.7%) relative to BC (4.3%, 4.2% and 1.3%) and N-CNS metastases (3.2%, 4.9% and 1.3%).

**Table 2:** Prevalence (%) of the genomic signatures HRD-gLOH, TMB, MSI, PDL1, PDL2 and PD-L1+ protein expression

	Site	% HRD-gLOH	% TMB-H	% MSI-H	%PDL1	%PDL2	% PDL1 +
**ALL**	local BC	33.70% (2573/7636)	5.04% (527/10464)	0.37% (39/10464)	1.57% (188/11988)	1.53% (183/11988)	54.25% (1092/2013)
	BCBM	**52.01%** (362/696)	**15.51%** (116/748)	**2.01%** (15/748)	**3.53%** (29/822)	**3.41%** (28/822)	38.73% (55/142)
	N-CNS	31.55% (3237/10260)	9.82% (1395/14210)	0.23% (32/14210)	1.12% (174/15516)	1.08% (168/15516)	26.21% (615/2346)
**ER+/HER2−**	local BC	31.48% (119/378)	3.41% (19/557)	0.36% (2/557)	0.94% (6/641)	0.78% (5/641)	40.00% (14/35)
	BCBM	**43.17%** (79/183)	**11.40%** (22/193)	1.04% (2/193)	0.94% (2/212)	0.47% (1/212)	23.08% (9/39)
	N-CNS	29.65% (126/425)	8.92% (51/572)	0.17% (1/572)	0.32% (2/634)	0.32% (2/634)	12.99% (10/77)
**ER+/HER2+**	local BC	19.57% (9/46)	2.78% (2/72)	0.00% (0/72)	1.19% (1/84)	1.19% (1/84)	42.86% (3/7)
	BCBM	26.67% (16/60)	9.68% (6/62)	0.00% (0/62)	1.52% (1/66)	1.52% (1/66)	27.27% (3/11)
	N-CNS	47.37% (18/38)	19.57% (9/46)	0.00% (0/46)	0.00% (0/49)	0.00% (0/49)	14.29% (1/7)
**ER−/HER2+**	local BC	32.26% (10/31)	8.70% (4/46)	0.00% (0/46)	1.75% (1/57)	1.75% (1/57)	100.00% (5/5)
	BCBM	33.33% (23/69)	**21.33%** (16/75)	2.67% (2/75)	0.00% (0/85)	0.00% (0/85)	57.14% (4/7)
	N-CNS	34.38% (11/32)	4.26% (2/47)	0.00% (0/47)	3.92% (2/51)	3.92% (2/51)	42.86% (3/7)
**ER−/HER2−**	local BC	52.25% (186/356)	2.70% (14/518)	0.19% (1/518)	3.42% (20/585)	3.59% (21/585)	50.00% (30/60)
	BCBM	**70.45%** (174/247)	**17.41%** (47/270)	**2.59%** (7/270)	**7.72%** (23/298)	**7.72%** (23/298)	48.33% (29/60)
	N-CNS	43.52% (84/193)	10.04% (27/269)	0.74% (2/269)	3.78% (11/291)	3.44% (10/291)	21.43% (6/28)

The significant alterations in brain metastases (BCBM) compared to local breast cancers (BC) are in bold letters. PDL1+ protein expression was lower in all the BMs compared to BCs except the ER-/HER2- BCBM subtype that is similar to BC and higher than non-central nervous system (N-CNS) metastases.

The prevalence of markers for immune checkpoint inhibition (ICI) in BCBM, tumor mutation burden (TMB; cutoff 10 mutations/Mb) and microsatellite instability (MSI), were also investigated^19,20^. 15.5% of BCBMs were TMB-High, 2.0% were MSI-High, 3.5% showed *PD-L1* (CD274) alterations (3.4% were amplification) and 3.4% had *PDCD1LG2* (PD-L2, CD273) amplification. All were significantly higher in the BCBM versus local BC and N-CNS groups (*p* < 0.0001 for all comparisons). TMB-High remained significant in ER-positive/HER2-negative (11.4%, *p* = 0.0002) and ER-negative/HER2-negative (17.4%, *p* < 0.0001) BCBMs compared to BCs but not in any other subtype.

PD-L1 positive expression as identified by IHC using the Ventana PD‐L1 SP142 assay^21,22^, was lower in BCBMs (38.7%) compared to BCs (54.3%) but higher than N-CNS (26.2%). PD-L1 protein expression according to subtypes was lower in BCBM than BC and higher than N-CNS (Table 2) with ER-positive/HER2-negative (23%), ER-positive/HER2-positive (27%), ER-negative/HER2-positive (57%). Of note, similar PD-L1 protein expression was observed in ER-negative/HER2-negative BCBMs (48.3%) compared to BCs (50.0%) but higher than in N-CNS (21.4%). Table 2 summarizes the percentage prevalence of the genomic signatures, of PD-L1/L2 alterations and PD-L1 positivity by IHC in the local BC, BCBM and N-CNS cohorts and within different receptor subtypes.

A subset of TMB-High BCBM samples (*n* = 18) tested positive for PD-L1 expression (12/18, 66.7%). Although PDL1-positivity was lower in TMB-High BCBMs than the local BCs (83/113, 73.5%), it was still higher than the PDL1-positivity in TMB-High N-CNS metastatic sites (84/236, 35.6%).

### Clinical correlations analysis and targetable genes

The majority of BCBMs had present at least one targetable gene (*PIK3CA*, *ERBB2*, *BRCA1/2*, *ARID1A*, *PARP1*, *CDKN2A/2B*), Fig. 1 and Table 1. In addition, many of these samples tested positive for one or more ICI markers (TMB-High, MSI-High, *PD-L1, PD-L2*, Table 2). These data highlight that a significant proportion of patients with BCBM might be eligible for targeted therapy and/or immunotherapy either as a single agent or in combination if such genomic data was available. Given this, the actionability of the targetable markers was further evaluated in the OncoKB database (www.oncokb.org)^23^. For example, the United States Food and Drug Administration (FDA)-approved drugs (Level 1) are available for alterations in *ERBB2, PIK3CA* for breast cancer and other biomarkers such as MSI-High, TMB-High for all solid tumors. There is clinical evidence (Level 3) that mutations in *AKT1*, *BRCA1*/*2*, *IDH1* can be targeted by Capivasertib, Olaparib or Talazoparib, Ivosidenib respectively whereas biological evidence (Level 4) links *CDK12* truncating mutations with Pembrolizumab-Nivolumab-Cemiplimab and *CDKN2A* oncogenic mutations with Palbociclib-Ribociclib-Abemaciclib. Gene alterations as identified in the BCBM cohort of this study with therapeutic drugs and level of evidence using the search terms ‘Breast cancer’, ‘CNS’ and ‘Glioma’ are summarized in Table 3. The percentage frequency of BCBM (by receptor status) where there is a targetable marker present, and a list of current clinical trials as identified in www.clinicaltrials.org are summarized in Supplementary Table 1.

**Table 3:** Actionability of targeted gene alterations

Level	Gene	Alterations	Cancer	Drugs (for therapeutic implications only)	% frequency in BCBM
1	ERBB2	Amplification	Breast Cancer	Ado-Trastuzumab Emtansine	24.57%
1				Lapatinib + Capecitabine, Lapatinib + Letrozole	
1				Margetuximab + Chemotherapy	
1				Neratinib, Neratinib + Capecitabine	
1				Trastuzumab + Pertuzumab + Chemotherapy	
1				Trastuzumab + Tucatinib + Capecitabine	
1				Trastuzumab Deruxtecan	
1				Trastuzumab, Trastuzumab + Chemotherapy	
1	PIK3CA	C420R, E542K, E545A, E545D, E545G, E545K, H1047L, H1047R, H1047Y, Q546E, Q546R		Alpelisib + Fulvestrant	30.17%
2		Oncogenic Mutations (excluding C420R, E542K, E545A, E545D, E545G, E545K, Q546E, Q546R, H1047L, H1047R and H1047Y)		Alpelisib + Fulvestrant	
3	AKT1	E17K		AZD5363	2.68%
3	BRCA1/2	Oncogenic Mutations		Olaparib	7.79%/5.47%
3				Talazoparib	
3	ERBB2	Oncogenic Mutations		Neratinib	24.57%
3	ESR1	Oncogenic Mutations		Fulvestrant	6.45%
1	NTRK1/2/3	Fusions	All Solid Tumors	Entrectinib, Larotrectinib	3.65%/ND
1	Other Biomarkers	MSI-High		Pembrolizumab	2.01%
1	Other Biomarkers	TMB-High		Pembrolizumab	15.51%
3	IDH1	R132	Glioma	Ivosidenib	0.0%
3	NTRK1/2/3	Fusions	All Solid Tumors	Repotrectinib	3.65%
4	ARID1A	Truncating Mutations		PLX2853	7.30%
4				Tazemetostat	
4	BRAF	G464, G469A, G469R, G469V, K601, L597		PLX8394	1.58%
4	CDK12	Truncating Mutations		Pembrolizumab, Nivolumab, Cemiplimab	4.01%
4	CDKN2A	Oncogenic Mutations		Palbociclib, Ribociclib, Abemaciclib	10.34%
4	EGFR	A289V, R108K, T263P	Glioma	Lapatinib	3.65%
4		Amplification			
4	FGFR1/2/3	Oncogenic Mutations		Debio1347, Infigratinib, Erdafitinib, AZD4547	10.46%/3.04%/0.61%
4	KRAS	Oncogenic Mutations	All Solid Tumors	Trametinib, Cobimetinib, Binimetinib	4.50%
4	MET	Fusions		Crizotinib	0.85%
4	MTOR	Oncogenic Mutations		Everolimus, Temsirolimus	0.49%
4	NF1	Oncogenic Mutations		Trametinib, Cobimetinib	8.64%
4	PTEN	Oncogenic Mutations		GSK2636771, AZD8186	16.67%
R1	NTRK1/3	G595R		Larotrectinib	3.65%
R2	NTRK1	G595R		Entrectinib	3.65%

Gene alterations with therapeutic drugs and level of evidence as identified in OncoKB (www.oncokb.org). 1: FDA-approved drugs, 2: Standard of care, 3: Clinical and 4: Biological evidence. R1/R2: Resistance.

## Discussion

Genomic profiling facilitated a deeper understanding of the evolutionary process of breast cancer and the development of metastasis by allowing the comparison of primary tumors with metastatic deposits^11–14,24^. This genomic characterization has resulted in the identification of somatic and germline genetic alterations leading to the development and implementation of targeted therapies^24,25^. It is known that the genomic landscape differs between primary and metastatic breast cancer with additional mutation burden and the acquisition of new driver mutations observed during the progression of breast cancer^26,27^, in addition to those that emerge as the result of treatment^28–30^. Given this, the acquisition and sequencing of metastatic disease is important^31^.

For BCBMs, the available genomic data is limited. Within three large genomic studies, which have characterized the genomic landscape of a total of 1985 MBCs, only 33 (1.7%) were BCBMs^11–13^. In a recently published systematic review utilizing data from 13 sequencing studies, we reported the commonly mutated genes identified in a total of 164 BCBMs^17^. A comparison of these data with those of extracranial disease demonstrated the differences between primary breast cancers and brain metastases and emphasized the importance of acquiring and analyzing tissue samples from patients’ brain metastases.

In this study, using the validated Foundation Medicine test and the largest BCBM cohort (*n* = 822) available to date (5 times larger than the data in the systematic review)^17^, we have investigated clinically-relevant genomic alterations present in BCBM and how they compare to the local BCs and/or N-CNS disease. The genomic differences seen in BCBM in comparison to local BCs and/or N-CNS may reflect the acquisition of additional mutations including new driver mutations during the metastatic process, or the clonal selection of cells able to seed BCBMs, though additional studies will be required to further confirm these hypotheses^26–30^.

Some of the identified alterations would lead to the consideration of specific therapeutic interventions if BC and N-CNS metastases genomic analyses were unable to reveal these alterations and patients had not yet been exposed to some of the available alteration-driven drugs. For instance, the *ERRB2* alterations would be potentially targetable with HER2-directed therapy. Tucatinib in combination with trastuzumab and capecitabine and trastuzumab deruxtecan have shown a high degree of intracranial activity in patients with HER2-positive brain metastases^32–34^. BCBMs harboring *ESR1* alterations could potentially be amenable to treatment with novel oral selective estrogen receptor downregulators such as elacestrant (EMERALD)^35^. Given the documented intracranial activity of the *CDK4/6* inhibitor, abemaciclib^36,37^ the combination of abemaciclib and elacestrant is currently being investigated in HR-positive, HER2-negative BCBM (NCT04791384). FGF/FGFR alterations are representing an emerging resistance pathway and therapeutic target in breast cancer^38^. Importantly, a recent study demonstrated that *FGFR* aberrations increased the incidence of BCBM, as well as being associated with a poorer prognosis in BC patients^39^. It has been also reported that *FGFR* aberrations together with *TP53* and *FLT1* genetic aberrations, and HER2-positivity were found that could predict BCBM^39^.

In this large cohort of BCBMs, we observed a high prevalence of homologous recombination repair alterations, including *BRCA1, RAD51B, BARD1*, and *PALB2* alterations, in addition to higher rates of HRD-gLOH. *PALB2* somatic alterations were also at a higher level identified in 2.7% of BCBMs in this study compared to 1.3% in BCs and N-CNS metastases. These would suggest the potential use of Poly [ADP-ribose] polymerase 1 (PARP) inhibitors or other DNA damaging agents such as platinum therapy^40,41^. Currently, the PARP inhibitors olaparib and talazoparib are licensed only for metastatic breast cancer with germline *BRCA1/2* alterations^42,43^. The OlympiAD and EMBRACA clinical trials of olaparib and talazoparib, respectively, showed a benefit of PARPi in metastatic breast cancer with germline BRCA1/2 pathogenic variants as compared to chemotherapy and in particular they demonstrated the benefit in those with stable intracranial disease at baseline^42,43^. Furthermore, the adjuvant OlympiAD trial found patients randomized to the olaparib arm had a numerically lower incidence of brain metastasis as compared to those treated with placebo^44^. These data in the metastatic and adjuvant setting provide evidence of the intracranial activity of PARP inhibitors. The efficacy of PARP inhibitors beyond germline *BRCA1/2* has been highlighted in a recent phase II study of talazoparib monotherapy which demonstrated activity in patients with germline *PALB2* pathogenic variants^45^, while TBCRC 048 has shown activity of olaparib in breast cancers with somatic *BRCA1/2* mutations^46^. These data taken together indicate that PARP inhibitors are a therapeutic strategy, which require exploration in patients with brain metastasis with HRD.

In addition to the high HRD-gLOH prevalence, several ICI biomarkers were also present at a high prevalence in this BCBM cohort. These included TMB-High, MSI-High, and PD-L1 (measured by IHC and by genomic amplification). TMB-High, an FDA-approved ICI biomarker in advanced cancer^47^, was observed in 15% of BCBM and over 20% of ER-negative/HER2-positive BCBMs, suggesting a broad population that may be potentially treated with ICI. ER-negative/HER2-negative BCBMs have also significantly high HRD-gLOH and MSI, whereas *PD-L1/L2* alterations were a distinctive feature of this subtype in BCBM. Genomic findings of increased frequency of MSH6 alterations, MSI-High, and higher rates of PDL1/2 amplification are consistent with an immune-activated phenotype. In the SAFIR02-BREAST IMMUNO trial, it was observed that CD274 gain/amplification could define a group of patients with high sensitivity to anti-PD-L1 immunotherapy^48^.

We recently showed that PD-L1 and CTLA4 transcripts were significantly higher in TN BCBMs, with CTLA4 expression also high in HER2-positive compared to estrogen receptor-positive BCBMs^49^. Studies of immunotherapy in the context of melanoma, non-squamous cell lung and leptomeningeal metastatic disease have provided proof of concept that immunotherapy can modulate intracranial disease and result in clinical benefit in intracranial disease^50–54^. In a recent phase 2 trial (NCT02886585) evaluating efficacy of pembrolizumab 37% of BCBM patients achieved an intracranial benefit with manageable toxicity. However, as the authors comment, this promising efficacy will need to be carefully balanced with the risk of toxicity and additional studies are required to enhance the therapeutic benefit of PD-1 blockade in rationally designed combinatorial regimens^55^. Given these data, the role of immunotherapy in appropriately biomarker selected breast cancer patients with BCBM should be explored. Based on our data, up to half (48.3%) of TN patients with BCBM could be eligible for immunotherapy with PDL1 inhibitors.

Finally, although large structural rearrangements were uncommon in BCBM, we identified a high prevalence (3.5%) of *CDK12* REs. The prevalence of *CDK12* alterations in BCBM requires further investigation given their association and possible involvement with ICIs. In advanced prostate cancer, a study identified *CDK12* mutant cases associated with elevated neoantigen burden and increased tumor T cell infiltration and summarized that *CDK12* inactivation defines a distinct class of metastatic castration-resistant prostate cancer that may benefit from ICI therapy^56^. A recent study analyzing a broad range of cancer types demonstrated that *CDK12* alterations are associated with the tandem-duplicator phenotype in cancer^57^. These data suggest that *CDK12* inactivation warrants further investigation as a pan-cancer biomarker for immunotherapy benefit.

The rarer, but still potentially actionable alterations as reported in BC^58,59^ are also enriched in the BCBM cohort (Supplementary Data 1). Given these data, the development of a BCBM umbrella trial, enrolled using comprehensive genomic profiling to test the efficacy of targeting these rare alterations should be considered. Such umbrella designs have been successfully used in the study of metastatic breast cancer (plasmaMATCH) and carcinomas of unknown primary (CUPSICO)^60,61^.

A key consideration in the development of any studies related to targeting CNS disease will be the ability of the agent to cross the blood-brain-barrier (BBB). The BBB is disrupted in CNS disease making access for any molecule easier. Furthermore, as highlighted above several agents including antibodies have been shown intracranial activity providing evidence that the disrupted BBB may not be an impediment^33,34,50,51^. While specific agents, such as the PI3K/mTOR inhibitor paxalisib have been designed to be brain penetrant^62^. The development of evidence in a systematic fashion regarding CNS penetrance within phase I studies should be considered for all novel agents.

The limitations of this study are that while it describes the largest dataset of BCBM it lacks paired primary and non-CNS metastatic tissue that could’ve have provided additional useful information in regard to the evolution of disease from primary cancer to CNS disease. However, this is common to other studies which have investigated the genomic landscape of non-CNS metastatic disease^11,12^. The samples profiled in this study were obtained during routine clinical care; given cases had to be sent for profiling this could have introduced bias based on access to funding for the profiling or the willingness of the healthcare team to request the assay. Many of the identified alterations have been also reported in smaller studies without this bias and inclusive of paired tissues^14,15^ as summarized in our previous systematic review^17^. Our current work highlights these alterations in a largest dataset supporting their importance. Finally, we lack complete clinicopathological characteristics and treatment information that would have provided additional information and insights. Nevertheless, the data from this large study reinforce the importance of acquiring metastatic material from the brain for comprehensive genomic profiling to enable rationale treatment decisions as well for the development and enrollment into genomically driven BCBM studies of targeted agents. For some therapies, such as ICIs and PARP inhibitors, testing of multiple biomarkers will likely be necessary to identify those patients who may derive clinical benefit. Given that not all patients undergo biopsy or resection of brain metastasis, in addition to the need to be able to sample from multiple lesions and at different time points, the use of ‘liquid biopsy’ to assess circulating tumor DNA (ctDNA) will be required. As it has been demonstrated that plasma is not an appropriate source for ctDNA derived from CNS disease, collection of cerebrospinal fluid will be required^63,64^. The feasibility of this collection is currently being assessed in a UK-based study (PRIMROSE-CSF (IRAS ID 286155). Going forward the approach may provide opportunities for longitudinal follow up of BCBM and guiding treatment decisions.

## Methods

### Patient cohort

This study consisted of distinct cohorts of 822 consecutive patients with BCBM, 11,988 local BC biopsies, and 15,516 non-central nervous system (N-CNS) metastatic biopsies (Supplementary Data 1, Prev all). The samples were collected during the course of routine clinical care by Foundation Medicine Inc (Boston, MA) and analyzed by genomic profiling between September 2012 and December 2020 as previously described^65,66^. Haematoxylin-Eosin (H&E) stained sections from each formalin fixed paraffin embedded (FFPE) tumor tissue sample were reviewed by a board-certified pathologist for the presence of adequate tumor (≥20% of nucleated cells are tumor cells) before sequencing. Receptor status was obtained by reviewing the accompanying pathology reports for hormone receptor status and ERBB2 (HER2) status from genomic profiling. The patients’ characteristics are presented in Supplementary Table 2.

### Targetable genomic profiling

Genomic profiling for all classes of alterations [short variants (SV; substitutions/insertions/deletions), copy number (CN; gain/loss) and rearrangements (RE; large structural aberrations including fusions)] in at least 324 genes was performed during routine clinical care using tumor-only hybrid capture-based next-generation sequencing in a Clinical Laboratory Improvement Amendments–certified laboratory (Foundation Medicine Inc, Boston, MA)^65,66^. Sequence analysis methods and validation of the CGP platform used in this study have been described previously by Frampton and colleagues^65^. Base substitution detection was performed using a Bayesian methodology, which enables the detection of somatic mutations at low mutant allele frequency (MAF) and increased sensitivity for mutations at hot‐spot sites through the incorporation of tissue‐specific prior expectations. Reads with mapping quality <25 were discarded, as were base calls with quality ≤2. Final calls were made at MAF of ≥5% (MAF ≥ 1% at hot spots) to avoid false‐positive calls, after filtering for strand bias (Fisher test, *p* < 0.001), read location bias (Kolmogorov–Smirnov test, *p* < 0.001), and presence in ≥two normal controls. To detect short insertions or deletions (indels), de novo local assembly in each targeted exon was performed using the De Bruijn approach. After read pairs were collected and decomposed, the statistical support for competing haplotypes was evaluated and candidate indels were aligned against the reference genome. Filtering of indel candidates was carried out as described for base substitutions^65^. Gene amplifications and homozygous deletions were detected by comparing complete chromosomal copy number maps to reference process‐matched, normal control samples, and gene fusions and rearrangements were detected by analysis of chimeric read pairs. CN alterations reported represent amplifications for oncogenes (CN > 6) and deep deletions (CN = 0) for tumor suppressors^66^. Only known or likely pathogenic alterations were included in the analysis, representing loss of function alterations (e.g. nonsense, frameshift, splice, deletion, truncation) in tumor suppressors or gain of function alterations (e.g. amplifications, fusions, activating missense mutations) in oncogenes. All events were manually curated based on the existing literature and public databases. Homologous recombination deficiency detected by genome-wide loss of heterozygosity (HRD-gLOH; cutoff 16%), high tumor mutation burden (TMB-High; cutoff 10 mutations/Mb), high microsatellite instability (MSI-High) were all called as previously described^18–20,32,66,67^. HRD-gLOH used a cut-off of 16% as reported in the ARIEL 3 trial in ovarian cancer^18^. Prevalence values were calculated in the real-world cohort and were not adjusted for factors like age, sex, grade, stage, or treatment status.

### PD‐L1 immunohistochemistry

Immunohistochemistry (IHC) for PD-L1 was performed using the Ventana PD‐L1 SP142 assay as described previously and was available for a subset of samples (1703 local BCs, 122 BCBMs and 1978 N-CNS)^21,22^. Briefly, the VENTANA SP142 CDx assay consists of the rabbit monoclonal anti-PD-L1 SP142 clone, the Opti-View DAB IHC detection kit, the Opti-View amplification kit stained on the VENTANA BenchMark ULTRA instrument using the staining protocol provided by the package insert and interpreted with the guidelines of the VENTANA interpretation guide (Foundation Medicine, Morrisville, NC)^21,22^. PD‐L1 staining was interpreted using the tumor‐infiltrating immune cell (IC) scoring method, where the IC score is the proportion of tumor area that is occupied by PD‐L1 staining ICs of any intensity per the VENTANA interpretation guide^22^.

### Actionability of targeted gene alterations

The actionability of the targetable gene alterations was evaluated in the OncoKB database (www.oncokb.org)^23^. The OncoKB database contains biological and clinical information about cancer. OncoKB uses a ‘Levels of Evidence’ system, assigning clinical actionability to individual mutational events where level 1 account for FDA-recognized biomarker predictive of response to an FDA-approved drug, level 2 for Standard of care biomarker predictive of response to an FDA-approved drug, levels 3 and 4 for Clinical and Biological evidence respectively supporting the biomarker as being predictive of response to a drug. R1 and R2 levels indicate Resistance biomarkers. The searches were performed utilizing the terms Breast cancer, CNS and Glioma/Glioblastoma. The term ‘CNS’ was present in the term ‘All solid tumors’ as presented in Table 3. The list of current clinical trials excluding untreated or uncontrolled CNS metastases but including treated and/or asymptomatic CNS was identified in www.clinicaltrials.org.

### Statistical analysis

Fisher’s exact test was used to examine the differences in the genomic landscape between the different cohorts. Of the 296 genes baited across all baitsets, we selected those with a prevalence of at least 0.5% in any of the comparative groups; *p*-values were adjusted for multiple comparisons using the Benjamini-Hochberg method for false discovery rate (FDR). FDR values < 0.1 were considered significant.

### Ethics approval and consent to participate

Approval for this study, including a waiver of informed consent and Health Insurance Portability and Accountability Act (HIPAA) waiver of authorization, was obtained from the Western Institutional Review Board (protocol #20152817). The Institutional Review Board granted a waiver of informed consent under 45 CFR § 46.116 based on review and determination that this research meets the following requirements: (i) the research involves no more than minimal risk to the subjects; (ii) the research could not practicably be carried out without the requested waiver; (iii) the waiver will not adversely affect the rights and welfare of the subjects. The study was performed in accordance with the Declaration of Helsinki.

## Supplementary information

Supplementary information file

Supplementary Data 1

Supplementary Data 2

## Data Availability

The sequencing data generated in this study is derived from clinical samples. The data supporting the findings of this study are provided within the paper and its supplementary files. All supplementary tables accompanying the different analyses and figures presented in this study are provided in Supplementary Data 1. Due to Health Insurance Portability and Accountability Act (HIPAA) (HIPAA) requirements, we are not consented to share individualized patient genomic data, which contains potentially identifying or sensitive patient information. Foundation Medicine is committed to collaborative data analysis, and we have well-established, and widely utilized mechanisms by which investigators can query our core genomic database of >600,000 de-identified sequenced cancers to obtain aggregated datasets.
