# Supplementary material for: Breast cancer brain metastases genomic profiling identifies alterations targetable by immune-checkpoint and PARP inhibitors
Source: NPJ Precis Oncol. 2024 Dec 20;8:282. doi: 10.1038/s41698-024-00761-0 (PMC11662007; doi:10.1038/s41698-024-00761-0)
Supplement: Supplementary file 1 — Supplementary information file [file 41698_2024_761_MOESM1_ESM.pdf]

Supplementary Table 1. Targeted therapy and/or immunotherapy in breast cancer brain metastases. The % frequency of BCBM targetable biomarkers by receptor status as identified in this study with a selection of current clinical trials. *Trials excluding untreated or uncontrolled CNS but including treated and/or asymptomatic CNS. **TMB % prevalence.

Altered Gene / Pathway	Targeted Therapy	Phase	Clinical Trials Identifier	ER+/HER2- BCBM	ER+/HER2+ BCBM	ER-/HER2+ BCBM	ER-/HER2- BCBM
**ERBB2/HER2 alterations**	Tucatinib, T-DM1	3	NCT03975647	2.83%	100%	100%	2.01%
	Pertuzumab,Trastuzumab	2	NCT02536339				
	Afatinib, T-DM1	2	NCT04158947				
	Cabozantinib, Trastuzumab	2	NCT02260531				
	Paxalisib, Trastuzumab	2	NCT03765983				
	Everolimus, Vinorelbine, Trastuzumab	2	NCT01305941				
	Pyrotinib, Vinorelbine	2	NCT03933982				
	GRN1005, Trastuzumab, 18F-FLT	2	NCT01480583				
	Neratinib, Capecitabine, T-DM1	2	NCT01494662				
	Afatinib, Vinorelbine	2	NCT01441596				
	Tucatinib, Capecitabine, Trastuzumab	2	NCT02614794				
	Trastuzumab deruxtecan	2	NCT04420598				
	68GaNOTA-Anti-HER2 VHH1	2	NCT03331601				
	Cabazitaxel, Lapatinib	2	NCT01934894				
	Afatinib, Targeted radiotherapy	1/2	NCT02768337				
	Trastuzumab deruxtecan	2	NCT04420598				
	Pyrotinib, Capecitabine, Radiotherapy	2	NCT04582968				
	Trastuzumab, Pertuzumab, TKIs	2	NCT04760431				
	Tucatinib, Pertuzumab, Trastuzumab	2	NCT05041842				
	Lapatinib Ditosylate, Radiotherapy	2	NCT01622868				
	Trastuzumab deruxtecan	2	NCT04752059				
	Trastuzumab deruxtecan	3	NCT04739761				
	ARX788	2	NCT05018702				
**ESR1 alterations**	Palbociclib	2	NCT02896335	16.98%	7.58%	ND	ND
	Palbociclib, AIs, Fulvestrant	2	NCT04256941				
	Abemaciclib, Stereotactic radiotherapy	1/2	NCT04923542				
	Abemaciclib, Elacestrant	1/2	NCT04791384				
**PIK3CA mutations**	Paxalisib	1	NCT04192981	38.21%	24.24%	36.47%	23.49%
	Paxalisib, Abemaciclib, Entrectinib	2	NCT03994796				
	Buparlisib, Capecitabine, Trastuzumab	2	NCT02000882				
**BRCA1/2 mutations**	Veliparib, Cisplatin	2	NCT02595905	5.19%/7.55%	ND	2.35%/2.35%	14.43%/6.04%
	Olaparib	3	NCT02032823				
	Olaparib, Durvulumab	1/2	NCT04711824				
	Olaparib, chemotherapy	3	NCT02000622*				
	Olaparib	3	NCT03286842				

**PDL1**	Pembrolizumab, Anti-HER2/3 Dendritic Cell Vaccine	0	NCT04348747	ND	1.52%	ND	7.72%
	Nivolumab, Stereotactic radiotherapy	1	NCT03807765				
	Atezolizumab, Stereotactic radiotherapy	2	NCT03483012				
	Pembrolizumab	1/2	NCT03449238				
	Atezolizumab, T-DM1	3	NCT04740918				
**PDL1/TMB****	Atezolizumab, Trastuzumab, Vinorelbine	2	NCT04759248	11.40%	9.68%	21.33%	17.41%
**HRD**	Niraparib, Dostarlimab	2	NCT04983745	43.17%	26.67%	33.33%	70.45%
	Olaparib	2	NCT03344965				
	Talazoparib	2	NCT02401347*				
**FGFR**	E7090	3	NCT04962867	21.7%	7.58%	10.59%	ND
**TP53+other gene mutations**	Flucytosine, Vocimagene Amiretrorepvec	1	NCT02576665	43.87%	54.55%	78.82%	90.60%
**VEGFA**	Bevacizumab, Etoposide,Cisplatin, WBRT	2	NCT02185352	ND	1.52%	3.53%	8.05%
**Multiple targets**	The MATCH Screening Trial	2	NCT02465060				

Supplementary Table 2. Patient demographics and baseline disease characteristics.

**Category**	**BC**	**BCBM**	**N-CNS**
Number of samples	11988	822	15516
Age median[IQR], y	55.0 [46.0-65.0]	54.0 [45.0-62.0]	59.0 [50.0-67.0]
**Sex %[n]**			
Male	1.018 [n=122]	0.608 [n=5]	1.044 [n=162]
Female	98.982 [n=11862]	99.392 [n=817]	98.956 [n=15352]
**Tumor type %[n]**			
breast carcinoma (nos)	16.008[n=1919]	87.713 [n=721]	80.833 [n=12542]
breast invasive ductal carcinoma (idc)	72.598 [n=8703]	11.071[n=91]	14.089 [n=2186]
breast invasive lobular carcinoma (ilc)	8.008[n=960]	0.608 [n=5]	4.189 [n=650]
breast metaplastic carcinoma	2.236 [n=268]	0.608 [n=5]	0.464 [n=72]
breast phyllodes tumour	0.375 [n=45]	0.0 [n=0]	0.199 [n=31]
breast ductal carcinoma in situ (dcis)	0.309 [n=37]	0.0 [n=0]	0.0 [n=0]
breast mucinous carcinoma	0.150 [n=18]	0.0 [n=0]	0.064 [n=10]
breast inflammatory carcinoma	0.117 [n=14]	0.0 [n=0]	0.064 [n=10]
breast lobular carcinoma in situ	0.067 [n=8]	0.0 [n=0]	0.045 [n=7]
breast carcinosarcoma	0.058 [n=7]	0.0 [n=0]	0.026 [n=4]
breast myoepithelial carcinoma	0.042 [n=5]	0.0 [n=0]	0.013 [n=2]
breast papillary carcinoma	0.033 [n=4]	0.0 [n=0]	0.013 [n=2]
**Tumour subtype %[n]**			
ER+/HER2-	5.3% [n=641]	25.8% [n=212]	4.1% [n=634]
ER+/HER2+	0.7% [n=84]	8.0% [n=66]	0.3% [n=49]
ER-/HER2+	0.5% [n=57]	10.3% [n=85]	0.3% [n=51]
ER-/HER2-	4.9% [n=585]	36.3% [n=298]	1.9% [n=291]
Unknown	88.6% [n=10621]	19.6% [n=161]	93.4% [n=14491]

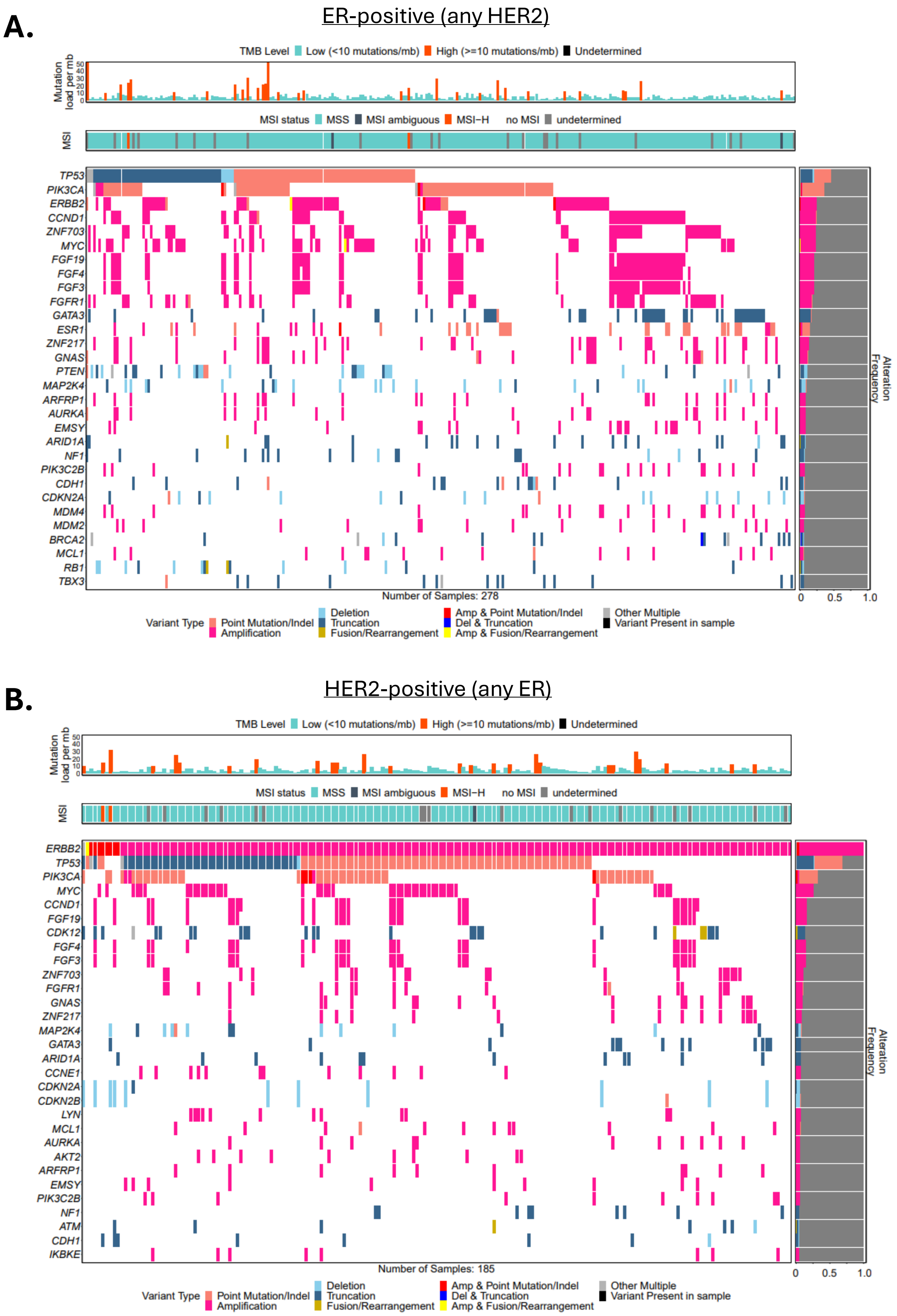

Supplementary Figure 1. Genomic Alterations in BCBM by receptor status. Tile-plots of genomic alterations in BCBMs classified as **A.** ER-positive (Any HER2 status) and **B.** HER2-positive (Any ER status). The different types of genomic variation are indicated by different colours. MSI and TMB for each subtype are also included in the plots.
